# Public health round-up

**DOI:** 10.2471/BLT.26.010926

**Published:** 2026-09-01

**One Health action plan extended**

The quadripartite collaboration on One Health, comprising the Food and Agriculture Organization of the United Nations, United Nations Environment Programme, World Health Organization and the World Organisation for Animal Health, has extended the implementation of the *One Health Joint plan of action* from its original 2022–2026 period to 2029. This extension provides countries with greater predictability to plan, implement and scale national One Health priorities, while enabling continued alignment among governments, partners and stakeholders. The plan will remain the shared framework for coordinated, multisectoral action to prevent, detect and respond to health risks at the human, animal, plant and ecosystem interface.

**Figure:**
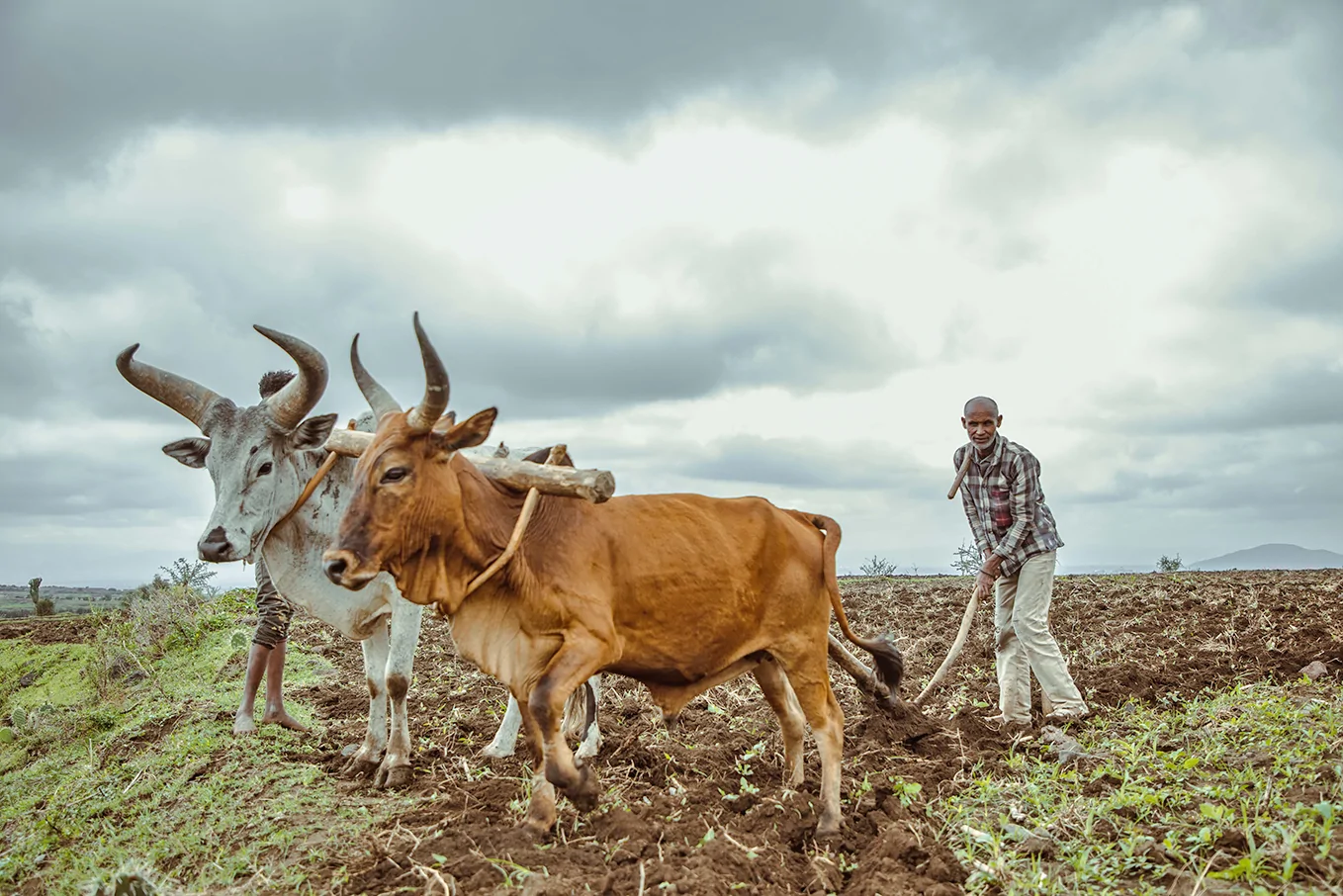
WHO/ Nitsebiho Asrat

## Climate, migration and health agenda

The World Health Organization (WHO) has launched a new global research agenda and action plan to address the health impacts of climate-related migration and displacement, bringing together more than 300 participants from over 75 countries. The initiative aims to strengthen the evidence base on how climate change affects population mobility, health outcomes and access to care.

As climate change increasingly contributes to migration and displacement, significant evidence gaps remain on how climate-related mobility affects health, well-being and access to health care, particularly for vulnerable populations.

“Closing research gaps is a matter of equity. To achieve universal health coverage, strengthen climate resilience and advance health equity, migrant and displaced populations must not be an afterthought, they must be part of the solution from the beginning,” said Santino Severoni, head of WHO’s special initiative on health and migration

Developed through WHO’s Global research prioritization exercise, the agenda identifies priority areas including universal health coverage, emergency preparedness, migration-sensitive data systems, mental health and the long-term health impacts of climate-related mobility.

By strengthening partnerships and supporting evidence-based decision-making, the roadmap aims to advance more resilient, climate-responsive and migrant-inclusive health systems worldwide. 

https://bit.ly/4gpkIps

## Community evidence for Bundibugyo virus disease

The World Health Organization (WHO) has received a 3 million United States dollars grant from Wellcome to strengthen the use of community-generated evidence in the response to the ongoing Bundibugyo virus disease outbreak. The funding will support the integration of social and behavioural insights alongside epidemiological, clinical and laboratory data, helping authorities make faster and more effective public health decisions.

Since the outbreak was declared a Public Health Emergency of International Concern in May 2026, WHO and partners have been conducting rapid community assessments in affected areas of the Democratic Republic of the Congo. These assessments capture how people perceive risk, access health care, respond to public health measures and experience the wider impacts of the outbreak.

Findings are already informing operational improvements, including stronger community surveillance, better access to health hotlines, expanded psychosocial support for health workers and clearer communication of laboratory results. The evidence has also identified ways to boost confidence in treatment services by reducing ambulance delays and improving patient reception capacities.

"Every outbreak is shaped not only by the pathogen, but also by how people understand risk, access care and respond to public health measures,” said Chikwe Ihekweazu, executive director of WHO’s health emergencies programme. “This investment is about making social analytics part of how outbreak intelligence works in practice. By integrating community-generated evidence throughout the response, we can build a more complete picture of the outbreak and make faster, more effective decisions that ultimately save lives."

As the Bundibugyo virus disease response continues, WHO and its partners are working to ensure that community-generated evidence becomes a routine component of outbreak intelligence, helping decision-makers respond more quickly, build trust and improve the effectiveness of public health interventions during this outbreak and future health emergencies.

https://bit.ly/4wu3exV

## Research4Life at 25

On July 21, WHO marked the 25th anniversary of Research4Life, a public-private partnership that provides researchers, educators, health professionals and policy-makers in low- and middle-income countries with free or low-cost access to scientific literature.

Launched by WHO in 2001 as a pilot initiative with six publishing partners, Research4Life has grown into one of the world's largest partnerships focused on equitable access to scientific publications. Today, it brings together more than 185 publishers and partner organizations, providing over 12 000 institutions in more than 120 countries with access to more than 250 000 journals, books and databases.

"What began as an effort to close the knowledge gap in health has grown into a global movement for research equity," said Sylvie Briand, WHO chief scientist. "For 25 years, Research4Life has helped ensure that researchers, educators, health professionals and policy-makers everywhere can access the evidence they need to improve lives and advance sustainable development."

In addition to improving access to scientific publications, Research4Life now supports researcher training, publishing skills and international collaboration. Through its 2030 strategy, the partnership aims to address persistent barriers to knowledge sharing, including publication costs that continue to limit the ability of many researchers to disseminate their work.

https://bit.ly/4xoEWa9

## Greener pharmaceuticals

WHO has launched the first global framework to help medicines regulators support the transition to lower-carbon pharmaceutical systems while maintaining standards of quality, safety, efficacy and equitable access.

The framework, *Towards a greener pharmaceuticals' regulatory highway*, outlines how regulators can use existing tools, including standards, scientific guidance, reliance mechanisms, digitalization and innovative regulatory pathways, to encourage the adoption of more sustainable technologies across the pharmaceutical lifecycle.

Health systems are estimated to account for around 5% of global greenhouse gas emissions, with pharmaceutical products contributing through manufacturing, packaging, transport, use and disposal. The new framework focuses on enabling wider uptake of existing decarbonization solutions while avoiding unnecessary regulatory burden, delays or barriers to access.

Priority actions include strengthening climate literacy among regulators, improving alignment on emissions measurement and reporting, expanding digital regulatory services, streamlining sustainability-related post-approval changes and promoting greater collaboration between regulatory authorities.

“As regulators, we are not passive actors in this system; we are architects of it,” said Delese Mimi Darko, director general of the African Medicines Agency. “By embedding sustainability into our regulatory frameworks, we can support innovation, strengthen local manufacturing and build more resilient health systems that ensure patients continue to have access to quality safe and efficacious medical products."

The framework is designed to support a just and inclusive transition, ensuring that sustainability efforts do not compromise access to medicines, particularly in low- and middle-income countries.

https://bit.ly/4wxdQfo

## New leishmaniasis treatment guidelines

WHO has issued updated treatment guidelines for visceral leishmaniasis, also known as kala-azar, and post-kala-azar dermal leishmaniasis, recommending shorter, safer and more effective treatment regimens for patients in eastern Africa and South-East Asia.

Leishmaniasis is one of the world's deadliest parasitic diseases, causing an estimated 50 000 to 90 000 cases annually. If left untreated, visceral leishmaniasis can be fatal, while post-kala-azar dermal leishmaniasis, a skin condition that may develop after treatment, can contribute to ongoing transmission and is often associated with stigma and social isolation.

“For too long, patients suffering from leishmaniasis have endured treatments nearly as punishing as the disease itself, especially in Africa. These new WHO guidelines mark a turning point,” said Daniel Ngamije Madandi, WHO director of malaria and neglected tropical diseases. “By recommending safer, shorter and more patient-friendly regimens, we are not just improving care; we are accelerating our fight to eliminate this devastating disease and offering renewed hope to communities across Africa and Asia.”

For the first time, WHO recommends sodium stibogluconate-free treatment options for eligible patients. New regimens include oral miltefosine combined with paromomycin for visceral leishmaniasis and post-kala-azar dermal leishmaniasis in eastern Africa, and shorter combination therapies using liposomal amphotericin B and miltefosine for post-kala-azar dermal leishmaniasis in South-East Asia. 

https://bit.ly/4xoHXat

**Cover photo**

Naomi Kembi and her daughter sit together outside their home in Kilifi County, Kenya. Naomi survived stage 3 cervical cancer and she now advocates for HPV vaccination. In November 2025, Kenya transitioned from a two-dose to a single-dose HPV vaccination schedule, following WHO SAGE recommendations.

**Figure:**
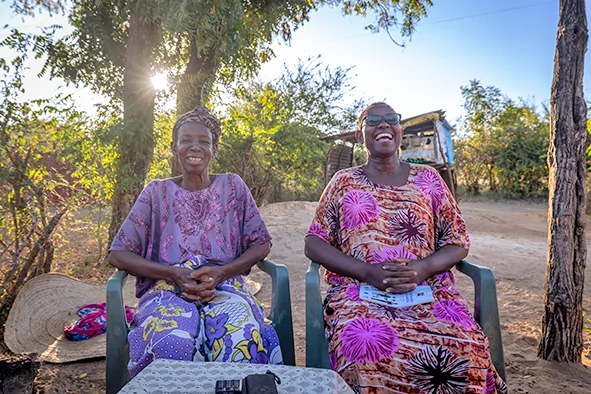
WHO/Genna Print

**Looking ahead**

1–30 September. World sexual and reproductive health month 2026. Virtual events. https://bit.ly/4coW6vA

16–18 September. Third meeting of the Global Initiative on AI for Health. https://bit.ly/4x06sKD

